# Electrochemical Activity and Corrosion Behavior of Ultrafine-Grained Ti-xMo Alloys Processed via Powder Metallurgy

**DOI:** 10.3390/ma19071431

**Published:** 2026-04-03

**Authors:** Katarzyna Arkusz, Kamila Pasik, Ewa Paradowska, Aleksandra Jędrzejewska, Mieczysław Jurczyk

**Affiliations:** 1 Department of Biomedical Engineering, Faculty of Engineering and Technology, University of Zielona Gora, 9 Licealna Street, 65-417 Zielona Gora, Poland; k.arkusz@iimb.uz.zgora.pl (K.A.); k.pasik@iimb.uz.zgora.pl (K.P.); e.paradowska@iimb.uz.zgora.pl (E.P.); a.jedrzejewska@iimb.uz.zgora.pl (A.J.); 2 The Doctoral School of Exact and Technical Sciences, University of Zielona Gora, 65-417 Zielona Gora, Poland

**Keywords:** titanium alloys, mechanical alloying, powder metallurgy, electrochemical corrosion, electrochemical activity

## Abstract

Titanium alloys are widely used for biomedical implants due to their favorable mechanical properties, corrosion resistance, and biocompatibility. However, the development of multifunctional implant materials requires not only structural stability but also controlled electrochemical responsiveness, an important property for electrochemical sensing. This study developed ultrafine-grained Ti–*x*Mo alloys (*x* = 28 and 31 wt.%) via mechanical alloying followed by powder metallurgy to investigate the effect of high Mo content on phase stability, corrosion behavior, and electrochemical sensing response. Both alloys exhibited predominantly β-phase microstructures, with β-phase fractions exceeding 93%, confirming effective stabilization at elevated Mo concentrations. Electrochemical tests conducted in 0.01 M PBS and Ringer’s solution revealed that pure Ti exhibited the highest impedance modulus and lowest corrosion current density, indicating superior passive film barrier properties. In contrast, high-Mo alloys showed reduced polarization resistance and increased charge-transfer contribution, associated with modifications in passive film defect chemistry and electronic properties induced by Mo enrichment. Among the investigated compositions, Ti-31 wt.% Mo demonstrated improved electrochemical stability compared to Ti-28 wt.% Mo, exhibiting lower corrosion current density and higher impedance values within the high-Mo regime. Cyclic voltammetry performed in 0.01 M PBS containing 1 mM K_3_[Fe(CN)_6_] confirmed enhanced heterogeneous electron-transfer capability for Mo-rich alloys relative to pure Ti. Overall, Ti-31 wt.% Mo provides a balanced combination of β-phase stabilization, moderate corrosion resistance, and improved electrochemical responsiveness potentially suitable for sensing interfaces.

## 1. Introduction

Titanium and its alloys have been extensively utilized in biomedical applications due to their exceptional combination of biocompatibility, corrosion resistance, and mechanical properties [1,2,3]. However, conventional titanium alloys such as Ti-6Al-4V, while widely used in orthopedic and dental implants, present several limitations including the release of potentially cytotoxic elements (Al and V), a high elastic modulus leading to stress shielding, and inadequate wear resistance [4,5]. These shortcomings have driven the development of new β-type titanium alloys with non-toxic alloying elements and improved mechanical properties [6].

Molybdenum has emerged as a particularly attractive β-stabilizing element for titanium alloys intended for biomedical applications. Mo is non-toxic, biocompatible, and effectively reduces the elastic modulus while maintaining adequate strength [7,8,9]. The addition of Mo lowers the β-transus temperature and stabilizes the β-phase at room temperature, resulting in a body-centered cubic crystal structure [10,11]. The β-phase exhibits a significantly lower elastic modulus than the α-phase, making β-rich Ti-Mo alloys more suitable for load-bearing implants by reducing stress-shielding effects [12].

The critical Mo concentration for complete β-phase stabilization in binary Ti-Mo alloys is approximately 10 wt.% [9]. However, higher Mo concentrations (15–35 wt.%) are often employed to ensure complete retention of the β-phase and further reduce the elastic modulus [1,7,8]. A systematic comparison of mechanical properties across different Mo concentrations reveals important trends and trade-offs. Table 1 summarizes the mechanical properties of Ti-Mo alloys with varying Mo content based on the reviewed studies.

Low-Mo-content alloys (5–10 wt.%) exhibit dual α + β phase microstructures with moderate mechanical properties. These alloys are easier to process and achieve higher densification, but offer only limited reductions in the elastic modulus [9,16,17]. Medium-Mo-content alloys (15–20 wt.%) represent a practical compromise, offering significant reductions in the elastic modulus (to ~78–84 GPa), good corrosion resistance, and adequate mechanical strength [9,13,18]. Ti-15Mo has been extensively studied and is considered a benchmark composition for biomedical applications [19]. High-Mo-content alloys (>20 wt.%) provide maximum β-phase stabilization and potential for further elastic modulus reduction [1]. However, these alloys face processing challenges, including reduced sinterability, potential brittleness, and higher material costs [16,20]. The benefits of high Mo content must be carefully weighed against these challenges for specific applications.

Recent research has focused on exploring high-Mo concentrations (>20 wt.%) in Ti-Mo alloys to maximize β-phase stability and further reduce the elastic modulus [1,18]. However, increasing Mo content presents challenges, including reduced densification during sintering, potential brittleness, and complex phase transformations [21,22]. Understanding the intricate relationships between the Mo concentration, processing parameters, microstructural evolution, mechanical properties, and electrochemical behavior is essential for optimizing these alloys for specific biomedical applications [23].

Beyond mechanical property modification, Mo significantly enhances corrosion resistance. Molybdenum enrichment in the passive oxide layer improves its stability and reduces ion release in physiological environments [3,9]. Increasing the Mo concentration enhances corrosion resistance through several mechanisms: (1) Mo enrichment in the passive film, (2) increased β-phase stability, (3) reduced galvanic coupling between phases, and (4) enhanced passive film repassivation kinetics [24]. Xu et al. [9] provided the most comprehensive study on the effect of Mo content (5–20 wt.%) on corrosion and tribocorrosion behavior. They reported that corrosion resistance improved progressively with increasing Mo content, with Ti-20Mo exhibiting the best performance [9]. The enhanced corrosion resistance was attributed to the formation of a more stable passive film with higher Mo oxide content [25]. For high-Mo-content alloys (>20 wt.%), Piechowiak et al. [1] demonstrated that Ti-23Mo, Ti-27Mo, and Ti-35Mo composites all exhibited good corrosion resistance. The study suggested that Mo contents above 20 wt.% ensures complete β-phase stabilization, eliminating galvanic coupling between the α and β phases, which can accelerate corrosion in dual-phase alloys [1]. The relationship between Mo content and corrosion resistance is not strictly linear at very high Mo concentrations [26,27]. Beyond a certain threshold (typically around 15–20 wt.%), the incremental improvement in corrosion resistance diminishes, suggesting that other factors such as the microstructure and surface condition become more important [9,14,28,29,30].

While the primary focus of Ti-Mo alloy research has been on structural implant applications [31], these materials also show promise for biosensing applications due to their excellent electrochemical properties [32,33] and biocompatibility [34,35]. Biosensors based on Ti-Mo alloys could enable real-time monitoring of physiological parameters in implantable devices. The stable passive oxide layer on Ti-Mo alloys provides an ideal platform for immobilizing biomolecules such as enzymes, antibodies, or DNA probes. The electrochemical properties of Ti-Mo alloys, including well-defined redox behavior and a low background current, are advantageous for electrochemical biosensing applications. Escobar et al. [15] evaluated the electrochemical behavior of Ti-18Mo-6Nb-5Ta alloy in PBS, demonstrating a stable electrochemical response with low corrosion current density. These characteristics are essential for biosensing applications, where stable baseline electrochemical behavior is required for accurate signal detection [13].

However, specific studies on biosensing capabilities and sensor performance of Ti-Mo alloys remain scarce, particularly for compositions with high Mo content exceeding 20 at.%. While such alloys are expected to exhibit enhanced β-phase stabilization and distinct electrochemical characteristics, their processing by powder metallurgy is challenging due to reduced diffusivity, potential segregation phenomena, and limited densification during sintering. Moreover, the electrochemical response of high-Mo Ti alloys in redox-active environments relevant to sensing applications has not been systematically investigated.

Despite extensive studies on Ti–Mo alloys, most investigations are limited to Mo contents below ~20 wt.% and focus primarily on corrosion resistance and mechanical properties. The electrochemical behavior of Ti–Mo alloys at higher Mo concentrations, particularly in the fully β-stabilized regime, remains insufficiently explored. Moreover, the combined effect of high Mo content and an ultrafine-grained microstructure obtained via mechanical alloying on electrochemical response has not been systematically addressed. Therefore, the present study aims to evaluate the structural evolution, corrosion resistance, and electrochemical sensing response of ultrafine-grained Ti-*x*Mo alloys (*x* = 28 and 31 wt.%) synthesized via mechanical alloying followed by powder metallurgy alloying. Particular attention is devoted to correlating β-phase stabilization and microstructural refinement with passive film characteristics and charge-transfer behavior in physiological media. By extending the compositional range toward high Mo contents and systematically investigating their electrochemical activity and corrosion behavior in the context of load-bearing biomedical applications, this work addresses an important knowledge gap in the development of multifunctional Ti–Mo alloys for next-generation implant systems.

## 2. Materials and Methods

### 2.1. Synthesis of Ti-xMo Samples

Mechanical alloying was performed at argon atmosphere (99.999% purity) using the SPEX 8000 Mixer Mill (Spex CertiPrep, Inc., Metuchen, NJ, USA). The total milling time was 48 h. The Ti and Mo powders were weighed, blended, and inserted into stainless steel vials in the glove box LabMaster 130 (MBraun, Garching, Germany), which was filled with an automatically controlled argon atmosphere (O_2_ < 2 ppm and H_2_O < 1 ppm). Powders of titanium (<45 µm, 99.9%, Alfa Aesar, Karlsruhe, Germany) and molybdenum (44 µm, 99.6%, Sigma Aldrich, Karlsruhe, Germany) were used. The weight ratio of hard steel balls to powder was 10:1. In the next step, the powder metallurgy process was applied to the produced powders. The conventional two-stage (cold pressure and sintering) approach was used to produce the bulk samples. In the cold pressing step, the precursors were inserted into a die and uniaxially pressed at 600 MPa. For sintering, the green compacts were placed in argon-filled quartz tubes, heated to 800 °C within 1 h, and held at this temperature for 30 min. The details of material production are introduced in our previous works [28,29,30]. The diameters and heights of the bulk samples were 8 mm and 4 mm, respectively.

### 2.2. Microstructural and Electrochemical Assessment

The crystallographic structure evolution of the samples during synthesis was studied at room temperature using X-ray diffraction (XRD) with a Panalytical Empyrean diffractometer equipped with CuKα (λ = 1.54056 Å) radiation (Almelo, The Netherlands). The lattice parameter estimation and phase quantitative analysis were based on Rietveld profile fitting performed in the Maud software version (2.99993). The applied approach involves simulating the diffraction pattern based on the analyzed structural model for: Ti(α) (ref. code 01-071-4632), Ti(β) (ref. code 01-074-7075), Mo (ref. code 01-071-4645). The density of the bulk sintered alloys was calculated by the Archimedes method. For the sample porosity measurement, the formula

(1) $$P=\left(1-\frac{\rho }{\rho_{th}}\right)\times 100\%$$

was used, where *ρ* is the density of the porous material, and *ρ*_th_ is its corresponding theoretical density calculated based on the rule of mixtures.

Phase morphology and elemental distribution were analyzed by scanning electron microscopy (SEM, JEOL JSM-7600F, Tokyo, Japan) and energy-dispersive X-ray spectroscopy (EDS, INCA, Oxford Instruments, Oxford, UK).

Electrochemical tests were carried out in a potentiostat/galvanostat AUTOLAB 302N Metrohm (Herisau, Switzerland). A three-electrode system was used: a saturated Ag/AgCl electrode as the reference electrode, a platinum counter electrode, and the sample of Ti-xMo as the working electrode. A 0.01 M PBS and Ringer’s solution was selected as testing media because the study was designed to evaluate the material performance in biosensing applications, blood-related measurements, and preliminary corrosion assessment for implant materials. Ti-*x*Mo specimens manufactured from virgin and reused powder were used as the working electrode.

Electrochemical measurements were performed in the following sequence: open circuit potential measurement for 1800 s, followed by electrochemical impedance spectroscopy (EIS) between 100 kHz and 0.1 Hz, with an AC amplitude of 10 mV and 10 points/decade. Potentiodynamic polarization curves were obtained by sweeping the electrode potential within ±250 mV relative to the open-circuit potential vs. Ag/AgCl, at a scan rate of 1.0 mV/s. The corrosion potentials (E_corr_), as well as the anodic (b_a_) and cathodic (b_c_) Tafel slopes, were determined by linear extrapolation of the respective branches of the polarization curves. The linear polarization resistance (R_p_) was calculated from the slope of the current–potential curve within ±2 mV around E_corr_. The corrosion current density (i_corr_) was subsequently determined using the Stern–Geary equation:

(2) $$R_{p}=\frac{b_{a}\cdot b_{c}}{2.303(b_{a}+b_{c})\cdot i_{corr}}$$

The corrosion rate (ν_corr_) was calculated according to the following equation:

(3) $$\nu_{corr}={3.17\cdot 10}^{-9}\frac{M}{nF\rho A}i_{corr}$$

where 3.17 × 10^−9^ is the conversion factor from cm/s to mm/year, M (g/mol) is the atomic weight of the sample, *n* is the number of electrons exchanged in the reaction, *ρ* (g/cm^3^) is the density of the sample, *F* (96,485 C/mol) is the Faraday constant, and A (cm^2^) is the surface area of the sample.

Cyclic voltammetry (CV) measurements were conducted in 0.01 M PBS to evaluate the dynamic electrochemical response and passive film stability of the Ti-*x*Mo alloys. To assess the heterogeneous electron-transfer behavior, CV was also performed in PBS containing 10 mM potassium ferricyanide (K_3_[Fe(CN)_6_]) as a reversible outer-sphere redox probe. The anodic and cathodic peak currents as well as peak-to-peak separation (ΔE_p_) were analyzed to characterize charge-transfer kinetics and the influence of Mo content on interfacial electrochemical activity.

All electrochemical tests were performed at room temperature, i.e., 25 °C. All tests were performed at least three times to ensure reproducibility.

### 2.3. Statistical Analysis

Statistical analysis was performed to evaluate differences between the studied materials (cp-Ti, Ti-28Mo, and Ti-31Mo). All data are presented as mean values ± standard deviation (SD). Due to unequal sample sizes and the potential violation of the assumption of homogeneity of variances, a variance-robust approach was applied. Overall differences between groups were assessed using Welch’s analysis of variance (Welch ANOVA). When statistically significant differences were detected, pairwise comparisons were performed using the Games–Howell post hoc test, which does not assume equal variances or equal sample sizes. A significance level of α = 0.05 was adopted for all statistical tests. Statistical analyses were carried out using Python (version 3.11.5) with the SciPy (version 1.11.3) and Statsmodels (version 0.14.0) libraries. Detailed results of the statistical analyses, including pairwise comparisons, are provided in the Supplementary Materials.

## 3. Results

Here, we examine how high Mo content (up to 31 wt.%) influences phase stability, passive film behavior, and electrochemical performance in mechanically alloyed Ti-Mo systems, thereby bridging structural biomaterial design with sensing-oriented electrochemical performance (Table 2).

**Figure 1:**
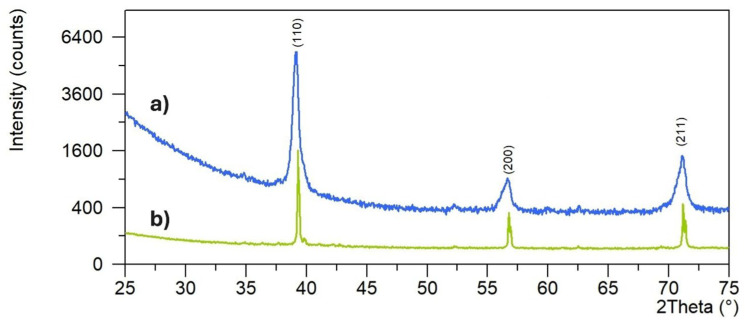
XRD diffractograms for the Ti-28 wt.% Mo (a) and Ti-31 wt.% Mo (b) alloys produced by mechanical alloying and powder metallurgy.

**Figure 2:**
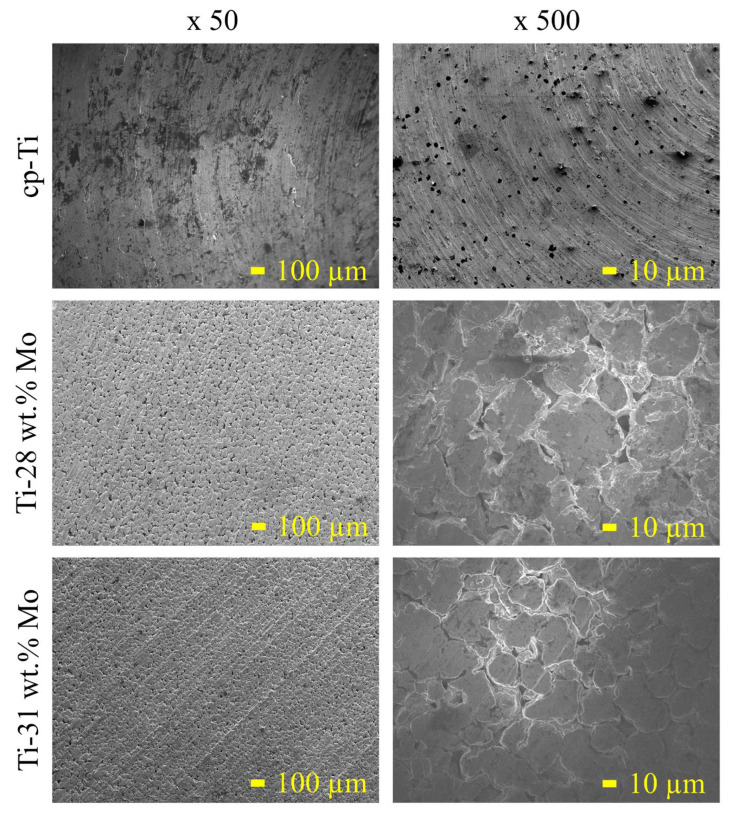
SEM micrographs of cp-Ti and Ti-*x*Mo (*x* = 28 and 31 wt.% Mo) samples.

The Ti-*x*Mo (*x* = 28 and 31 wt.%) alloys were obtained by mechanical alloying and powder metallurgy methods. The changes in the crystal structure during the milling process were studied earlier [30]. After 48 h of mechanical alloying, the Ti and Mo powders are almost amorphous, and the crystallite sizes calculated by the application of the W-H (UDM) approach were 16 and 18 nm for 28 and 31 wt.% of Mo content in Ti-Mo alloy, respectively. During the MA process, the phase transition from Ti(α) to Ti(β) is observed. Mechanically alloyed Ti-Mo powder compositions were finally cold pressed and sintered at temperatures of 800 °C for 0.5 h in argon. Figure 1 shows XRD spectra of the synthesized bulk alloys. In addition to the main Ti(β) phase, a small amount of Ti(α) is detected—see Table 3. The synthesized bulk Ti-Mo alloys show a porous microstructure (Figure 2).

Figure 2 presents SEM micrographs of cp-Ti and Ti-*x*Mo alloys (*x* = 28 and 31 wt.% Mo). The microstructure of cp-Ti is compact and morphologically uniform, without visible secondary phases at the microscale. This refined morphology results from severe plastic deformation and repeated cold welding fracture events occurring during high-energy mechanical alloying, as previously reported for Ti-based systems processed under similar conditions [17,28].

For Ti-28Mo, the microstructure remains homogeneous, and no discrete Mo-rich agglomerates or residual elemental particles are observed. This indicates effective solid-state diffusion and chemical homogenization during milling and sintering. Similar behavior has been reported for mechanically alloyed Ti-Mo systems, where extended milling promotes the dissolution of Mo into the Ti matrix and suppresses elemental segregation [9,16]. The lack of visible phase contrast at this scale suggests substantial stabilization of the β-phase, which is expected at Mo contents approaching or exceeding the critical concentration for β-retention.

A similar morphology is observed for Ti-31Mo. Despite the higher Mo content, no microcracking or intermetallic precipitation is detected. This observation is significant, because high Mo additions are often associated with reduced sinterability and increased brittleness [16,28]. The microstructural continuity observed here confirms that the selected mechanical alloying parameters (48 h milling, 10:1 BPR) were sufficient to obtain uniform alloy formation even at high Mo concentrations.

**Figure 3:**
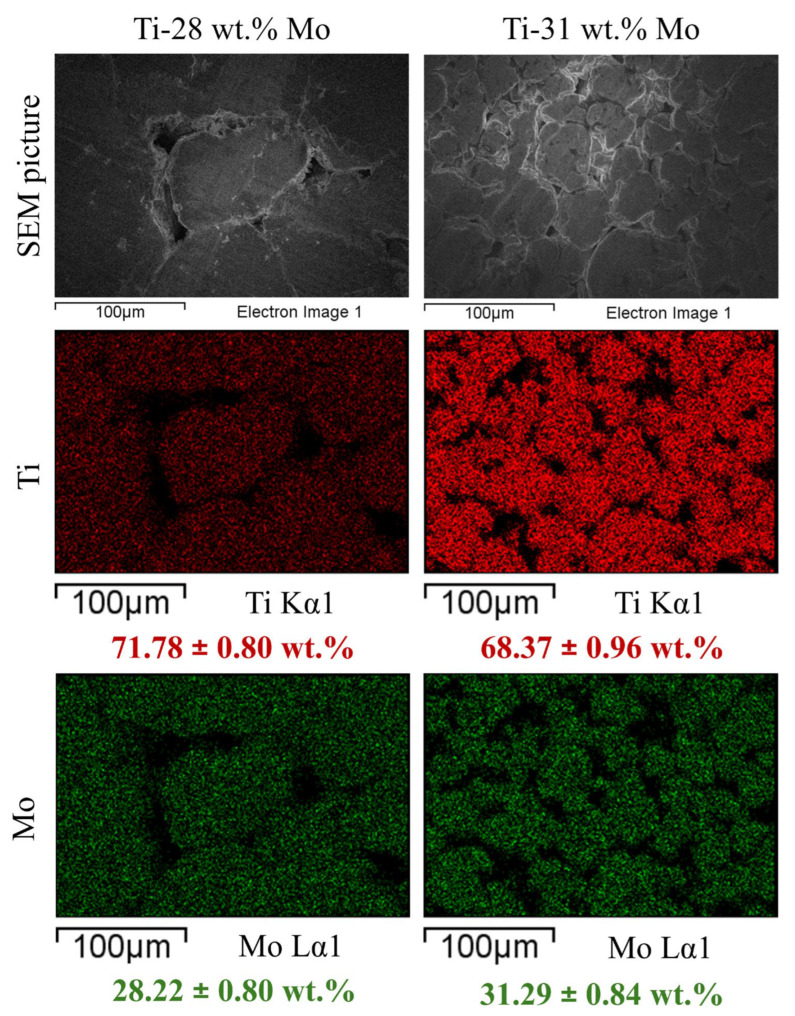
EDS elemental mapping of the Ti-28Mo and Ti-31Mo alloys produced by mechanical alloying and powder metallurgy (all results in wt.%).

Figure 3 shows SEM micrographs and EDS elemental maps of the Ti-28Mo and Ti-31Mo alloys. EDS elemental mapping confirms the presence and spatial distribution of Ti and Mo in both materials. The Ti and Mo signals are distributed relatively uniformly within the analyzed areas, indicating effective alloying during the mechanical alloying and sintering processes. For the Ti-28Mo alloy, the measured concentrations are 71.78 ± 0.80 wt.% for Ti and 28.22 ± 0.80 wt.% for Mo. In the Ti-31Mo alloy, the corresponding values are 68.37 ± 0.96 wt.% for Ti and 31.29 ± 0.84 wt.% for Mo. The obtained compositions are consistent with the nominal alloy compositions.

**Figure 4:**
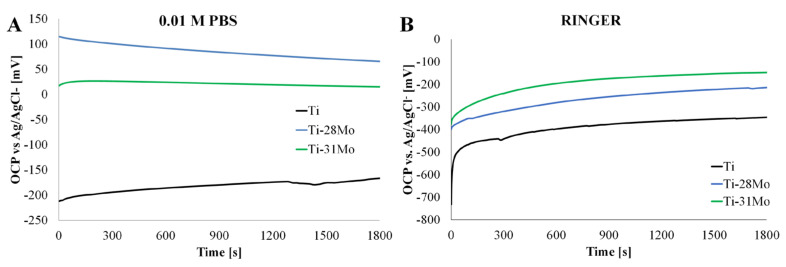
Open-circuit potential of cp-Ti and Ti-*x*Mo (*x* = 28 and 31 wt.%) measured during immersion for 1800 s at room temperature in: (**A**) 0.01 M PBS; (**B**) Ringer’s solution.

Figure 4 presents the evolution of the open-circuit potential (OCP) of Ti-*x*Mo alloys during 1800 s immersion in PBS (Figure 4A) and Ringer’s solution (Figure 4B), with steady-state values summarized in Table 4.

In PBS, cp-Ti stabilizes at −167 ± 25 mV after an initial positive shift related to the formation and growth of a TiO_2_ passive layer, commonly reported for titanium in physiological electrolytes [36]. In contrast, the Ti-28Mo and Ti-31Mo alloys reach more noble steady-state potentials of 66 ± 62 mV and 15 ± 33 mV, respectively. These differences were statistically significant (Welch ANOVA, *p* < 0.01, Table S1), with post hoc analysis confirming that cp-Ti differs from both Ti-Mo alloys, while no significant difference was observed between Ti-28Mo and Ti-31Mo (Table S2). The shift in OCP toward more positive values after Mo addition indicates changes in the surface electrochemical equilibrium, related to the modification of the passive layer and β-phase stabilization. A similar trend is observed in Ringer’s solution. Ti-31Mo reaches the most positive OCP (72 ± 4 mV), followed by Ti-28Mo (51 ± 23 mV) and cp-Ti (−65 ± 12 mV). Although overall differences were significant (*p* < 0.01, Table S1), pairwise comparisons revealed limited statistical separation, with a significant difference observed only between cp-Ti and Ti-28Mo (Table S2). Mo-containing alloys also stabilize faster, which suggests a more rapid establishment of the interfacial equilibrium.

Positive shifts in OCP with increasing Mo content were previously reported for Ti-Mo alloys containing up to 10–20 wt.% Mo in simulated body fluids and NaCl solutions [9,32]. Xu et al. [9] confirmed that Mo addition promotes more noble corrosion potentials, which was attributed to β-phase stabilization and Mo enrichment in the passive oxide layer. Similarly, Capela et al. [37] observed improved corrosion potential values for Ti-Mo alloys in chloride-containing electrolyte. The present results extend these observations to substantially higher Mo concentrations (28–31 wt.%). The continued positive shift in OCP indicates that Mo remains electrochemically active in modifying the surface thermodynamic equilibrium even at elevated alloying levels. The literature data confirm that Mo incorporation may lead to the formation of mixed Ti-Mo oxides and ${\mathrm{Mo}}^{6+}$ species within the passive layer [25], which can influence the anodic dissolution kinetics and shift the corrosion potential toward more noble values. The OCP characterizes the thermodynamic tendency for corrosion under open-circuit conditions and does not directly quantify the corrosion rate. Nevertheless, the systematic positive shift in OCP with increasing Mo content indicates that high-Mo Ti-Mo alloys exhibit a modified interfacial electrochemical equilibrium associated with β-phase stabilization and changes in the passive layer composition.

**Figure 5:**
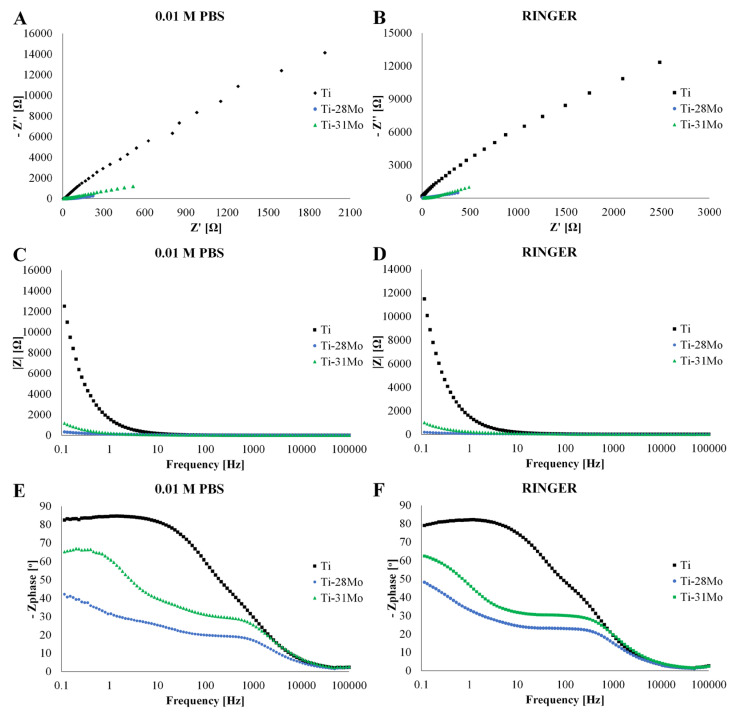
EIS analyses were performed to measure the electrical properties of cp-Ti and Ti-*x*Mo (*x* = 28 and 31 wt.%) samples in 0.01 M PBS (**A**,**C**,**E**) and Ringer’s (**B**,**D**,**F**) solution at room temperature as Nyquist (**A**,**B**), Bode magnitude (**C**,**D**), and Bode phase angle (**E**,**F**) plots.

The electrochemical impedance response of Ti-*x*Mo alloys in PBS and Ringer’s solution is presented in Figure 5, and the corresponding low-frequency parameters (0.1 Hz) are summarized in Table 4. Statistical analysis using Welch ANOVA confirmed significant differences between the studied groups in both electrolytes (*p* < 0.001 for all parameters). Subsequent Games–Howell post hoc tests were performed to identify pairwise differences.

In PBS, cp-Ti exhibits the highest impedance modulus (|Z| = 14,214 ± 1381 Ω) and a phase angle of −81.9 ± 1.3°, indicating dominant capacitive behavior and the presence of a compact and highly resistive passive film. The large semicircle observed in the Nyquist plot confirms high polarization resistance and limited charge-transfer contribution at the alloy/electrolyte interface. This behavior is consistent with the barrier properties of TiO_2_ films formed spontaneously on commercially pure titanium in physiological electrolytes [32,33]. In contrast, Ti-28Mo shows a pronounced reduction in impedance (|Z| = 447 ± 32 Ω) and a lower phase angle of −58.9 ± 0.8°. Ti-31Mo exhibits intermediate values (|Z| = 1113 ± 183 Ω; −56.2 ± 7.1°). These differences were statistically significant between all groups for |Z|, Z′ and −Z″ (*p* < 0.01, Table S3). For Zphase, cp-Ti differed significantly from both Ti-Mo alloys (*p* < 0.05), whereas no statistically significant difference was observed between Ti-28Mo and Ti-31Mo (*p* > 0.05, Table S4). The lower impedance modulus and phase angle indicate deviation from ideal capacitive behavior and a higher contribution of charge-transfer processes. Similar trends were reported for β-stabilized Ti-Mo alloys, where Mo addition modifies the composition of the passive film and increases its electronic conductivity [9,24,25]. A similar relation is observed in Ringer’s solution. Cp-Ti shows the highest impedance and phase angle (|Z| = 15,330 ± 1339 Ω; −78.7 ± 0.6°), while Ti-28Mo exhibits the lowest impedance and phase angle (|Z| = 535 ± 23 Ω; −45.6 ± 1.1°). Ti-31Mo again shows intermediate values (|Z| = 1255 ± 208 Ω; −65.1 ± 4.2°). Statistical analysis confirmed significant differences between all materials for |Z| and −Z″ (*p* < 0.05, Table S5), including between Ti-28Mo and Ti-31Mo. For Z′, significant differences were observed between cp-Ti and both Ti-Mo alloys (*p* < 0.001, Table S6), as well as between Ti-28Mo and Ti-31Mo (*p* < 0.05). In the case of Zphase, cp-Ti differed significantly from both Ti-Mo alloys (*p* < 0.05), whereas no significant difference was found between Ti-28Mo and Ti-31Mo (*p* > 0.05). The lower phase angle of Ti-28Mo suggests a higher resistive contribution and a less homogeneous passive layer compared to Ti and Ti-31Mo.

Interfacial impedance characteristics are critical in electrochemical systems and play an important role in electrochemical sensing. High impedance and near-ideal capacitive behavior, as observed for cp-Ti, provide a low background current and stable baseline conditions, which are important for long-term signal reproducibility. However, very high barrier resistance may limit heterogeneous electron-transfer kinetics required for redox-mediated sensing. The reduced impedance modulus and lower phase angle observed for Ti-Mo alloys indicate enhanced interfacial charge-transfer capability. The literature reports that Mo incorporation into Ti passive layers leads to the formation of mixed Ti-Mo oxides and modifies the defect chemistry of the passive layer, resulting in altered semiconducting properties and increased electronic transport through the oxide layer [9,25]. Such changes facilitate electron exchange between the electrode surface and redox-active species, which is crucial for amperometric biosensing systems. Therefore, cp-Ti exhibits superior barrier properties, while Ti-31Mo shows a more balanced electrochemical response with moderate impedance and measurable charge-transfer contribution. This combination is favorable for implantable biosensors, where passive stability and electrochemical responsiveness are required.

**Figure 6:**
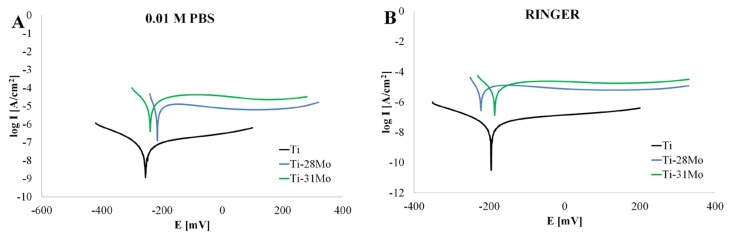
Potentiodynamic polarization curves of cp-Ti and Ti-*x*Mo (28 and 31 wt.%) samples recorded in: (**A**) 0.01 M PBS; (**B**) Ringer’s solution.

The potentiodynamic polarization curves of Ti-xMo alloys recorded in PBS and Ringer’s solutions are shown in Figure 6, and the corresponding electrochemical parameters derived from Tafel extrapolation are summarized in Table 5. Statistical analysis (Welch ANOVA with Games–Howell post hoc test) revealed that the observed differences depend on the electrochemical parameter, with significant effects primarily for polarization resistance and selected kinetic descriptors.

In PBS, cp-Ti exhibits the lowest corrosion current density (I_cor_ = 0.16 ± 0.04 µA) and the highest polarization resistance (R_p_ = 454,637 ± 38,445 Ω·cm^2^), indicating effective protection by the passive layer. The low corrosion rate (0.0031 ± 0.0006 mmpy) confirms high stability of the TiO_2_ passive layer in buffered physiological electrolyte, consistent with the previously reported behavior of commercially pure Ti [32,33]. Ti-31Mo exhibits a rapid increase in corrosion current (i_corr_ = 9.18 ± 1.4 µA) and a significant decrease in polarization resistance (R_p_ = 1492 ± 380 Ω·cm^2^). These results indicate a reduction in passive film barrier properties. Ti-28Mo shows improved behavior compared to Ti-31Mo, with lower I_cor_ (4.3 ± 1.68 µA) and higher R_p_ (1902 ± 296 Ω·cm^2^), although the corrosion resistance remains lower than for cp-Ti. These trends were statistically supported for R_p_ (*p* < 0.01, Table S7), where cp-Ti differed significantly from both Ti-Mo alloys, while no significant difference was observed between Ti-28Mo and Ti-31Mo. In contrast, no statistically significant differences were found for corrosion current density and the corrosion rate, despite apparent numerical trends (Table S8). The non-monotonic trend in the high-Mo range indicates that corrosion behavior is influenced not only by Mo content but also by phase constitution and passive film characteristics. A similar tendency is observed in Ringer’s solution. Cp-Ti maintains the lowest corrosion current (0.046 ± 0.0036 µA) and highest polarization resistance (842,271 ± 70,201 Ω·cm^2^), confirming stable passive behavior. Ti-31Mo again exhibits the highest corrosion current (4.73 ± 0.01 µA) and lowest polarization resistance (2054 ± 780 Ω·cm^2^), whereas Ti-28 Mo shows intermediate behavior (I_cor_ = 4.21 ± 1.03µA; R_p_ = 2122 ± 163 Ω·cm^2^). The corrosion rates follow the same tendency. Statistically, no significant differences were observed for corrosion potential, whereas significant differences were confirmed for R_p_, i_corr_ and the corrosion rate (*p* < 0.01, Table S9). Post hoc analysis indicated that cp-Ti differs significantly from Ti-31Mo, while no significant differences were detected between the Ti-Mo alloys (Table S10).

Polarization behavior provides additional insight into interfacial reactivity in electrochemical systems. Materials with very low I_cor_ and high R_p_, such as cp-Ti, provide excellent long-term stability and minimal background Faradaic processes, which are desirable for baseline signal stability. However, limited interfacial charge-transfer activity may reduce sensitivity in redox-mediated detection systems. The higher corrosion current and lower polarization resistance observed for high-Mo alloys indicate enhanced electrochemical activity at the electrode/electrolyte interface. The literature data show that Mo incorporation modifies the passive film composition and may increase its electronic conductivity or defect density [9,24,25]. These modifications can facilitate electron-transfer processes between the electrode surface and electroactive species. In amperometric and voltammetric biosensing systems, moderate interfacial reactivity can increase signal amplitude and improve electrochemical response.

Among the investigated alloys, Ti-31Mo exhibits more favorable behavior than Ti-28Mo, with lower I_cor_ and higher R_p_, while maintaining higher electrochemical activity relative to cp-Ti. A comparison with clinically used titanium alloys provides additional context for the obtained results. Ti-6Al-4V typically exhibits corrosion current densities in the order of 10^−7^–10^−6^ A/cm^2^ and polarization resistance in the range of 10^4^–10^5^ Ω·cm^2^ in physiological environments [38,39,40], while β-type alloys such as Ti-15Mo are generally characterized by stable passive behavior and improved corrosion resistance [18,19,24,27]. In this study, cp-Ti shows the highest polarization resistance, confirming its superior barrier properties. In contrast, the high-Mo Ti–Mo alloys exhibit significantly lower polarization resistance and higher corrosion current density, indicating a shift toward increased interfacial electrochemical activity. Relative to Ti-6Al-4V, the investigated alloys therefore do not provide improved corrosion resistance but instead display enhanced charge-transfer capability. This distinction is critical for application-oriented interpretation. Rather than maximizing corrosion resistance, high-Mo Ti–Mo alloys exhibit a different functional profile, combining passive stability with measurable electrochemical activity. Such behavior is not advantageous for conventional load-bearing implants, but may be beneficial for electrochemical and biosensing applications requiring controlled interfacial reactivity.

**Figure 7:**
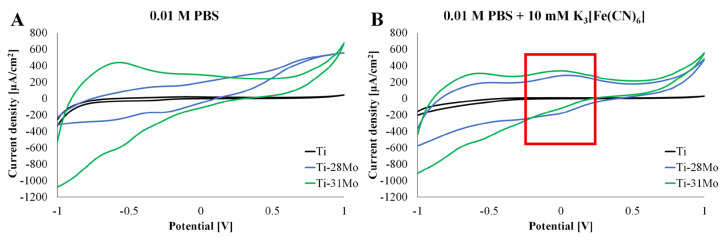
Cyclic voltammetry recorded in: (**A**) in 0.01 M PBS and (**B**) 0.01 M PBS with 1 mM potassium ferricyanide (K_3_[Fe(CN)_6_]) carried out using cp-Ti and Ti-xMo (*x* = 28 and 31 wt.%) samples as working electrodes and Ag/AgCl as reference electrode.

Figure 7 presents cyclic voltammograms recorded in 0.01 M PBS (Figure 7A) and 0.01 M PBS (Figure 7B) containing 1 mM K_3_[Fe(CN)_6_] using Ti-xMo alloys as a working electrode. In PBS without redox mediator (Figure 7A), all Ti-Mo alloys exhibit predominantly featureless voltammograms with no clearly resolved anodic or cathodic peaks, indicating the absence of significant Faradaic reactions within the scanned potential window. The current response is mainly capacitive and is associated with charging and discharging of the electrochemical double layer and the passive oxide film. Cp-Ti shows the lowest background current density, consistent with its high impedance modulus and near-ideal capacitive behavior observed in EIS (Figure 5). In contrast, Ti-28Mo and Ti-31Mo exhibit moderately elevated background currents, correlating with their reduced impedance and lower polarization resistance, which suggests increased interfacial conductivity or defect-mediated transport within the passive film of Mo-rich alloys.

In PBS containing 1 mM K_3_[Fe(CN)_6_] (Figure 7B), the current response increases compared to PBS alone. However, despite the presence of capacitive background currents, anodic and cathodic peaks characteristic of the Fe(CN)_6^3−^_/Fe(CN)_6^4−^_ redox couple are observable, although not sharply defined. Broad redox-like features in the central potential region indicate kinetically hindered electron transfer due to the passive oxide layer. Cp-Ti displays the lowest current and faintest redox features, consistent with its highly resistive TiO_2_ passive film and high charge-transfer resistance in EIS and polarization measurements. The literature reports that TiO_2_ films act as wide-bandgap semiconductors with limited conductivity, which restrict outer-sphere electron-transfer kinetics [32,33]. Ti-28Mo and Ti-31Mo show increased current density and more pronounced redox-like features compared to cp-Ti, indicating relatively enhanced interfacial electron-transfer capability. This behavior is consistent with the lower impedance modulus and reduced phase angle observed in EIS measurements. Mo incorporation into the passive layer is reported to modify its defect structure and electronic properties, potentially increasing electronic conductivity through the formation of mixed Ti-Mo oxides [9,25]. Between the two Mo-containing alloys, Ti-31Mo exhibits a more symmetric and stable voltammetric response, suggesting improved interfacial uniformity. CV results indicate that Mo enrichment enhances electrochemical responsiveness relative to pure Ti. Although distinct reversible redox peaks are not sharply resolved, the increased current responses observed for high-Mo alloys suggest improved electron-transfer capability, which is an important parameter in electrochemical sensing systems.

## 4. Conclusions

Ti-xMo alloys (x = 28 and 31 wt.% Mo) were successfully fabricated by mechanical alloying and powder metallurgy, resulting in homogeneous microstructures and effective β-phase stabilization at high Mo contents. Electrochemical measurements in PBS and Ringer’s solution demonstrated that pure Ti exhibits the highest impedance modulus, lowest corrosion current density, and superior passive film stability. In contrast, high-Mo alloys showed reduced polarization resistance and increased interfacial charge-transfer contribution. Within the high-Mo regime, Ti-31Mo demonstrated improved electrochemical stability compared to Ti-28Mo.

Cyclic voltammetry using the ferri/ferrocyanide redox probe indicated enhanced interfacial electrochemical responsiveness for Mo-rich alloys compared to pure Ti. However, the redox features remained partially masked by capacitive contributions of the passive oxide layer, suggesting that heterogeneous electron transfer is still kinetically limited by surface passivation.

Overall, Ti-31Mo provides a balanced combination of structural homogeneity, moderate corrosion resistance, and increased interfacial electrochemical activity. These characteristics indicate its potential as a structural platform for multifunctional biomedical systems combining mechanical support with electrochemical functionality. Nevertheless, for optimized performance in electrochemical applications, additional surface modification strategies (e.g., nanostructuring, conductive coatings, or functional interlayers) would likely be required to enhance electron-transfer kinetics and signal resolution.

## Figures and Tables

**Table 1:** Mechanical properties of Ti-Mo alloys as a function of Mo content.

Mo Content [wt.%]	Vickers Hardness HV	Young’s Modulus [GPa]	Key Findings	Ref.
5–10	195–240	85–95	Dual α + β phase, moderate strength	[9]
12–15	-	78–84	Good balance of properties, widely studied	[13,14]
18–20	285	79	Enhanced corrosion resistance	[9,15]
23–27	503–332	126–134	Ultrafine grain, good corrosion resistance	[1]
35	446	148	Complete β-stabilization, challenges in processing	[1]

**Table 2:** An equivalent weight and density of Ti-xMo alloys.

Alloy	Equivalent Weight [g·eq^−1^]	Density [g·cm^−3^]
Ti	47.87	4.500
Ti-28 wt.% Mo	61.34	6.953
Ti-31 wt.% Mo	62.78	7.226

**Table 3:** Phase amounts determined by the Rietveld method and lattice constants of the β-phase.

Alloy	Ti(β) [%]	Ti(α) [%]
Ti-28 wt.% Mo	95.93	4.07
Ti-31 wt.% Mo	93.23	6.77

**Table 4:** Electrochemical parameters obtained from the electrochemical impedance spectroscopy measurement (OCP—open-circuit potential, |Z|—impedance modulus, Z′—real part of impedance, −Z″—imaginary part of impedance, −Zphase—phase angle of impedance) measured at 0.1 Hz.

Alloy	OCP [mV]	|Z| [Ω]	Z′ [Ω]	−Z″ [Ω]	−Zphase [°]
**0.01 M PBS**					
Ti	−167 ± 25	14,214 ± 1381	1960 ± 196	14,075 ± 1400	81.9 ± 1.3
Ti-28 wt.% Mo	66 ± 62	447 ± 32	230 ± 11	383 ± 31	58.9 ± 0.8
Ti-31 wt.% Mo	15 ± 33	1113 ± 183	612 ± 136	920 ± 178	56.2 ± 7.1
**RINGER**					
Ti	65 ± 12	15,330 ± 1339	3015 ± 415	15,030 ± 1284	78.7 ± 0.6
Ti-28 wt.% Mo	72 ± 4	535 ± 23	336 ± 87	406 ± 24	45.6 ± 1.1
Ti-31 wt.% Mo	51 ± 23	1255 ± 208	522 ± 106	1138 ± 202	65.1 ± 4.2

**Table 5:** Corrosion parameters of cp-Ti and Ti-xMo (*x* = 28 and 31 wt.%) samples recorded in 0.01 M PBS and Ringer’s solution (E_cor_—corrosion potential, I_cor_—corrosion current, R_p_—linear polarization resistance).

Alloy	E_cor_ [mV]	I_cor_ [µA]	R_p_ [Ω·cm^2^]	Corrosion Rate [mmpy]
**0.01 M PBS**				
Ti	−246 ± 11	0.16 ± 0.04	454,637 ± 38,445	0.0031 ± 0.0006
Ti-28 wt.% Mo	−212 ± 6	4.3 ± 1.68	1902 ± 296	0.1469 ± 0.0728
Ti-31 wt.% Mo	−253 ± 19	9.18 ± 1.4	1492 ± 380	0.2776 ± 0.0424
**RINGER**				
Ti	−200 ± 9	0.046 ± 0.0036	842,271 ± 70,201	0.00091 ± 0.00007
Ti-28 wt.% Mo	−220 ± 6	4.21 ± 1.03	2122 ± 163	0.129 ± 0.0317
Ti-31 wt.% Mo	−298 ± 15	4.73 ± 0.01	2054 ± 780	0.143 ± 0.0002

## Data Availability

The raw data supporting the conclusions of this article will be made available by the authors on request.

## Supplementary Materials

The following supporting information can be downloaded at: https://www.mdpi.com/article/10.3390/ma19071431/s1. Table S1. Results of Welch ANOVA for OCP measurements of cp-Ti and Ti-xMo (28 and 31 wt.%) samples recorded in 0.01 M PBS and Ringer’s solution. Table S2. Statistical analysis (Games–Howell post hoc) for OCP measurements of cp-Ti and Ti-xMo (28 and 31 wt.%) samples recorded in 0.01 M PBS and Ringer’s solution. Table S3. Results of Welch ANOVA for EIS measurements of cp-Ti and Ti-xMo (28 and 31 wt.%) samples recorded in 0.01 M PBS solution. Table S4. Statistical analysis (Games–Howell post hoc) for EIS measurements of cp-Ti and Ti-xMo (28 and 31 wt.%) samples recorded in 0.01 M PBS solution. Table S5. Results of Welch ANOVA for EIS measurements of cp-Ti and Ti-xMo (28 and 31 wt.%) samples recorded in Ringer’s solution. Table S6. Statistical analysis (Games–Howell post hoc) for EIS measurements of cp-Ti and Ti-xMo (28 and 31 wt.%) samples recorded in Ringer’s solution. Table S7. Results of Welch ANOVA for corrosion parameters of cp-Ti and Ti-xMo (28 and 31 wt.%) samples recorded in 0.01 M PBS solution. Table S8. Statistical analysis (Games–Howell post hoc) for corrosion parameters of cp-Ti and Ti-xMo (28 and 31 wt.%) samples recorded in 0.01 M PBS solution. Table S9. Results of Welch ANOVA for corrosion parameters of cp-Ti and Ti-xMo (28 and 31 wt.%) samples recorded in Ringer’s solution. Table S10. Statistical analysis (Games–Howell post hoc) for corrosion parameters of cp-Ti and Ti-xMo (28 and 31 wt.%) samples recorded in in Ringer’s solution.

## References

[1] Piechowiak D., Miklaszewski A., Makuch-Dziarska N. (2021). The ultrafine-grain yttria-stabilized zirconia reinforced β-titanium matrix composites. Metals.

[2] Bălțatu M.S. (2020). Titanium-Based Alloys for Biomedical Applications.

[3] Tulinski M., Jurczyk M.U., Arkusz K., Nowak M., Jurczyk M. (2025). Nanotechnology for biomedical applications: Synthesis and properties of Ti-based nanocomposites. Nanomaterials.

[4] Abdelrhman Y., Gepreel M., Kobayashi M., Okano S. (2019). Biocompatibility of new low-cost (α + β)-type Ti-Mo-Fe alloys for long-term implantation. Mater. Sci. Eng. C.

[5] Valko M., Morris H., Cronin M.T.D. (2005). Metals, toxicity and oxidative stress. Curr. Med. Chem..

[6] Gummadi S.K., Kundu S., Chatterjee S. (2023). A review on titanium and titanium alloys with other metals for biomedical applications prepared by powder metallurgy techniques. Mater. Today Proc..

[7] Macías J.J., Aguilar C., Araya I., Castro F., Rojas P.A. (2024). In situ fabrication of Ti-xNb alloys by conventional powder metallurgy. Coatings.

[8] Awad N.K., Elshazly E.A., Fayyad A., Hassan A.M., Abdel-Jaber G.T., Nady A. (2024). Role of Mo and Zr additions in enhancing the behavior of new Ti–Mo alloys for implant materials. Met. Mater. Int..

[9] Xu W., Lu X., Zhang B., Liu C., Lv S., Yang S. (2020). Effects of Mo content on corrosion and tribocorrosion behaviours of Ti-Mo orthopaedic alloys fabricated by powder metallurgy. Corros. Sci..

[10] Xu W., Tian S., Lv S., Lu X., Yan M. (2019). Effects of Mo contents on the microstructure, properties and cytocompatibility of the microwave sintered porous Ti-Mo alloys. Mater. Sci. Eng. C.

[11] Hu Y., Zhao Y., Lu Y., Zhang Y., Wang H., Qiao J. (2023). Systematic study of (TiZr)xNby(TaMo)z medium entropy alloys for biomedical implants. J. Mater. Res. Technol..

[12] Sochacka P., Miklaszewski A., Jurczyk M. (2017). The influence of Mo content on phase transformation in Ti-Mo alloys. Arch. Metall. Mater..

[13] Awad A.H., Aly H.A., Saood M. (2023). Physical, mechanical, and corrosion properties of Ti–12Mo and Ti–15Mo alloys fabricated by elemental blend and mechanical alloying techniques. Mater. Chem. Phys..

[14] Yehia H.M., El-Tantawy A., Ghayad I.M., Eldesoky A.S., Elkady O.A. (2020). Effect of zirconia content and sintering temperature on the density, microstructure, corrosion, and biocompatibility of the Ti–12Mo matrix for dental applications. J. Mater. Res. Technol..

[15] Escobar J.D., Hernández J.P., Poblano-Salas C.A., Ruiz O., Soto D., Cabrera J.M. (2023). urface modified β-Ti-18Mo-6Nb-5Ta (wt%) alloy for bone implant applications: Composite characterization and cytocompatibility assessment. J. Funct. Biomater..

[16] Lee E.B., Han M.K., Kim B.J., Song H.J., Park Y.J. (2014). Effect of molybdenum on the microstructure, mechanical properties and corrosion behavior of Ti alloys. Int. J. Mater. Res..

[17] Gao Z., Luo H., Li Q., Wan Y. (2012). Preparation and characterization of Ti-10Mo alloy by mechanical alloying. Metallogr. Microstruct. Anal..

[18] Xu W., Xiao M., Lv S., Yagi K., Lu X., Yan M. (2018). Preparation and bioactive surface modification of the microwave sintered porous Ti-15Mo alloys for biomedical application. Sci. China Mater..

[19] Terynková A., Kozlík J., Bartha K., Chráska T., Stráský J. (2018). Ultra-fine grained Ti-15Mo alloy prepared by powder metallurgy. Mater. Sci. Forum.

[20] Gupta R.K., Kumar V.A., Mathew C. (2022). Influence of Mo Content on the Microstructure and Mechanical Properties of Ti6AlxMo Alloy. Key Eng. Mater..

[21] Guo Y., Chen Y., Chen C., Wang L., Liu J., Huang X. (2023). Ti-Mo-Zr alloys for bone repair: Mechanical properties, corrosion resistance, and biological performance. J. Mater. Res. Technol..

[22] Sutowo C., Ariyani N., Zulfia A. (2020). Microstructures, mechanical properties, and corrosion behavior of novel multi-component Ti-6Mo-6Nb-xSn-xMn alloys for biomedical applications. AIMS Mater. Sci..

[23] Liu B., Wang Z., Liu F., Xiu Z., Zhang M., Zhang Y. (2021). Microstructural evolution and biocompatibility of porous and low-cost Ti–Mo and Ti–Mo–Fe alloys. J. Nanoelectron. Optoelectron..

[24] Zhang S., Cui N. (2024). Enhancing passivation behaviors and wear resistance of biomedical Ti-15Mo alloy via {332} twinning pre-tension and aging. Coatings.

[25] Rodrigues A.V., Guastaldi A.C. (2018). Ti-Mo alloys: Corrosion study in solutions simulating commercial gels. J. Polym. Sci. Eng..

[26] Bouchemel H., Benchettara A. (2014). Corrosion behavior of a new Ti–3Mo alloy in simulated body fluid for biomedical applications. Arab. J. Sci. Eng..

[27] Rodrigues A., Oliveira N.T.C., Santos M.L.D., Guastaldi A.C. (2015). Electrochemical behavior and corrosion resistance of Ti-15Mo alloy in naturally aerated solutions containing chloride and fluoride ions. J. Mater. Sci. Mater. Med..

[28] Sochacka P., Miklaszewski A., Kowalski K., Jurczyk M. (2019). Influence of the processing method on the properties of Ti-23 at.% Mo alloy. Metals.

[29] Sochacka P., Jurczyk M.U., Kowalski K., Wildstein P.K., Jurczyk M. (2021). Ultrafine-grained Ti-31Mo-type composites with HA and Ag, Ta_2_O_5_ or CeO_2_ addition for implant applications. Materials.

[30] Sochacka P., Miklaszewski A., Jurczyk M. (2019). Development of type Ti-x at.% Mo alloys by mechanical alloying and powder metallurgy: Phase evolution and mechanical properties (10 ≤ x ≤ 35). J. Alloys Compd..

[31] Mareci D., Chelariu R., Gordin D., Romas M., Sutiman D., Gloriant T. (2010). Effect of Mo content on electrochemical behaviour of TiMo alloys for dental applications. Mater. Corros..

[32] Oliveira N.T.C., Aleixo G.T., Caram R., Guastaldi A.C. (2007). Development of Ti-Mo alloys for biomedical applications: Microstructure and electrochemical characterization. Mater. Sci. Eng. A.

[33] Sung B.S., Park T.E., Yun Y.H. (2015). Microstructures and electrochemical behavior of Ti-Mo alloys for biomaterials. Adv. Mater. Sci. Eng..

[34] Yehia H.M., El-Tantawy A., Elkady O.A., Ghayad I.M., Daoush W.M. (2024). Fabrication and characterization of Ti–12Mo/xAl_2_O_3_ bio-inert composite for dental prosthetic applications. Front. Bioeng. Biotechnol..

[35] Sandu A.V., Baltatu M., Vizureanu P., Sandu A., Florido-Suarez M. (2019). Characterization and mechanical properties of new TiMo alloys used for medical applications. Materials.

[36] Arkusz K., Pasik K., Nowak M., Jurczyk M. (2024). Structural, electrical and corrosion properties of bulk Ti–Cu alloys produced by mechanical alloying and powder metallurgy. Materials.

[37] Capela M.V., Acciari H.A., Capela J.M.V., Carvalho T.M., Melin M.C.S. (2008). Repeatability of corrosion parameters for titanium–molybdenum alloys in 0.9% NaCl solution. J. Alloys Compd..

[38] Gudić S., Vrsalović L., Nagode A., Gudić M., Žulj V. (2021). Electrochemical Behaviour of Ti and Ti-6Al-4V Alloy in Phosphate Buffered Saline Solution. Materials.

[39] Xu G., Wu R., Luo K., Lu J. (2022). Effects of heat treatment on hot corrosion behavior of directed energy deposited In718/316L functionally graded material. Corros. Sci..

[40] Jáquez-Muñoz J.M., Gaona-Tiburcio C., Méndez-Ramírez C.T., Baltazar-Zamora M.Á., Estupinán-López F., Bautista-Margulis R.G., Cuevas-Rodríguez J., Flores-De los Rios J.P., Almeraya-Calderón F. (2023). Corrosion of Titanium Alloys Anodized Using Electrochemical Techniques. Metals.

