# Supplementary material for: Electrochemical Activity and Corrosion Behavior of Ultrafine-Grained Ti-xMo Alloys Processed via Powder Metallurgy
Source: Materials (Basel). 2026 Apr 3;19(7):1431. doi: 10.3390/ma19071431 (PMC13074602; doi:10.3390/ma19071431)
Supplement: Supplementary file 1 [file materials-19-01431-s001.zip › materials-4214208-supplementary.pdf]

SUPPLEMENTARY

Electrochemical Activity and Corrosion Behavior of Ultrafine-Grained Ti-xMo Alloys Processed via Powder Metallurgy

Katarzyna Arkusz [1], Kamila Pasik [1,2], Ewa Paradowska [1], Aleksandra Jędrzejewska [1] and Mieczysław Jurczyk [1,*]

[1] Department of Biomedical Engineering, Faculty of Engineering and Technology, University of
Zielona Gora, 9 Licealna Street, 65-417 Zielona Gora, Poland; k.arkusz@iimb.uz.zgora.pl (K.A.); k.pasik@iimb.uz.zgora.pl (K.P.); e.paradowska@iimb.uz.zgora.pl (E.P.); a.jedrzejewska@iimb.uz.zgora.pl (A.J.)

[2] The Doctoral School of Exact and Technical Sciences, University of Zielona Gora, 65-417 Zielona Gora, Poland

* Correspondence: m.jurczyk@iimb.uz.zgora.pl

Table S1. Results of Welch ANOVA for OCP measurements of cp-Ti and Ti-xMo (28 and 31 wt.%) samples recorded in 0.01M PBS and Ringer's solution.

Parameter	F (df1, df2)	p-value	Significance
**OCP (PBS)**	F(2, 3.70) = 35.01	0.00393	Significant
**OCP (Ringer)**	F(2, 2.01) = 205.68	0.00471	Significant

Table S2. Statistical analysis (Games–Howell post hoc) for OCP measurements of cp-Ti and Ti-xMo (28 and 31 wt.%) samples recorded in 0.01M PBS and Ringer's solution.

Comparison	Mean difference	p-value	Significance
	**0.01M PBS**		
**Ti vs Ti-28Mo**	-208.75	0.0111	Significant
**Ti vs Ti-31Mo**	-175.07	0.00250	Significant
**Ti-28Mo vs Ti-31Mo**	33.68	0.411	Not significant
	**RINGER**		
**Ti vs Ti-28Mo**	-137.98	1.83×10^{-4}	Significant
**Ti vs Ti-31Mo**	-112.53	0.149	Not significant
**Ti-28Mo vs Ti-31Mo**	25.45	0.536	Not significant

Table S3. Results of Welch ANOVA for EIS measurements of cp-Ti and Ti-xMo (28 and 31 wt.%) samples recorded in 0.01M PBS solution.

Parameter	F (df1, df2)	p-value	Significance
**|Z|**	F(2, 4.92) = 132.69	5.21×10^{-5}	Significant
**Z′**	F(2, 4.09) = 100.22	3.34×10^{-4}	Significant
**–Z″**	F(2, 4.21) = 109.90	2.31×10^{-4}	Significant
**–Zphase**	F(2, 3.91) = 177.51	1.46×10^{-4}	Significant

Table S4. Statistical analysis (Games–Howell post hoc) for EIS measurements of cp-Ti and Ti-xMo (28 and 31 wt.%) samples recorded in 0.01M PBS solution.

Parameter	Comparison	Mean difference	p-value	Significance
**Z**	Ti vs Ti-28Mo	13821.72	5.70×10^{-4}	Significant
	Ti vs Ti-31Mo	13067.85	6.21×10^{-4}	Significant
	Ti-28Mo vs Ti-31Mo	-753.87	6.27×10^{-4}	Significant
**Z′**	Ti vs Ti-28Mo	1684.55	5.85×10^{-4}	Significant
	Ti vs Ti-31Mo	1367.46	1.02×10^{-4}	Significant
	Ti-28Mo vs Ti-31Mo	-317.09	0.0422	Significant
**−Z″**	Ti vs Ti-28Mo	13753.12	6.03×10^{-4}	Significant
	Ti vs Ti-31Mo	13124.64	5.84×10^{-4}	Significant
	Ti-28Mo vs Ti-31Mo	-628.48	0.0136	Significant
**−Zphase**	Ti vs Ti-28Mo	23.27	3.48×10^{-4}	Significant
	Ti vs Ti-31Mo	21.39	0.0333	Significant
	Ti-28Mo vs Ti-31Mo	-1.88	0.769	Not significant

Table S5. Results of Welch ANOVA for EIS measurements of cp-Ti and Ti-xMo (28 and 31 wt.%) samples recorded in Ringer's solution.

Parameter	F (df1, df2)	p-value	Significance
**|Z|**	F(2, 4.85) = 191.95	2.41×10^{-5}	Significant
**Z′**	F(2, 5.22) = 70.26	1.69×10^{-4}	Significant
**−Z″**	F(2, 4.81) = 200.88	2.34×10^{-5}	Significant
**−Zphase**	F(2, 3.64) = 28.54	0.00597	Significant

Table S6. Statistical analysis (Games–Howell post hoc) for EIS measurements of cp-Ti and Ti-xMo (28 and 31 wt.%) samples recorded in Ringer's solution.

Parameter	Comparison	Mean difference	p-value	Significance
**Z**	Ti vs Ti-28Mo	14637.90	1.49×10^{-4}	Significant
	Ti vs Ti-31Mo	13917.30	1.89×10^{-4}	Significant
	Ti-28Mo vs Ti-31Mo	-720.61	0.0126	Significant
**Z′**	Ti vs Ti-28Mo	2694.12	6.07×10^{-4}	Significant
	Ti vs Ti-31Mo	2518.05	7.39×10^{-4}	Significant
	Ti-28Mo vs Ti-31Mo	-176.07	0.0577	Not significant
**−Z″**	Ti vs Ti-28Mo	14451.17	1.31×10^{-4}	Significant
	Ti vs Ti-31Mo	13730.56	1.74×10^{-4}	Significant
	Ti-28Mo vs Ti-31Mo	-720.61	0.0130	Significant
**−Zphase**	Ti vs Ti-28Mo	29.73	0.053	Not significant
	Ti vs Ti-31Mo	13.42	0.0016	Significant
	Ti-28Mo vs Ti-31Mo	-16.31	0.143	Not significant

Table S7. Results of Welch ANOVA for corrosion parameters of cp-Ti and Ti-xMo (28 and 31 wt.%) samples recorded in 0.01M PBS solution.

Parameter	F (df1, df2)	p-value	Significance
E_{cor}	F(2, 3.32) = 14.41	0.0230	Significant
J_{cor}	F(2, 1.78) = 11.92	0.0933	Not significant
**Rp**	F(2, 3.20) = 173.70	5.46×10^{-4}	Significant
**Corrosion rate**	F(2, 1.78) = 10.32	0.105	Not significant

Table S8. Statistical analysis (Games–Howell post hoc) for corrosion parameters of cp-Ti and Ti-xMo (28 and 31 wt.%) samples recorded in 0.01M PBS solution.

Parameter	Comparison	Mean difference	p-value	Significance
E_{cor}	Ti vs Ti-28Mo	-34.00	0.0198	Significant
	Ti vs Ti-31Mo	7.33	0.507	Not significant
	Ti-28Mo vs Ti-31Mo	41.33	0.0213	Significant
J_{cor}	Ti vs Ti-28Mo	-4.14×10^{-6}	0.178	Not significant
	Ti vs Ti-31Mo	-1.15×10^{-5}	0.0453	Significant
	Ti-28Mo vs Ti-31Mo	-7.37×10^{-6}	0.0869	Not significant
**Rp**	Ti vs Ti-28Mo	452,734.66	0.00239	Significant
	Ti vs Ti-31Mo	453,386.57	0.00238	Significant
	Ti-28Mo vs Ti-31Mo	651.91	0.164	Not significant
**Corrosion rate**	Ti vs Ti-28Mo	-0.144	0.219	Not significant
	Ti vs Ti-31Mo	-0.350	0.0453	Significant
	Ti-28Mo vs Ti-31Mo	-0.206	0.113	Not significant

Table S9. Results of Welch ANOVA for corrosion parameters of cp-Ti and Ti-xMo (28 and 31 wt.%) samples recorded in Ringer's solution.

Parameter	F (df1, df2)	p-value	Significance
E_{cor}	F(2, 2.16) = 4.08	0.185	Not significant
J_{cor}	F(2, 1.53) = 2.61×10^{5}	5.68×10^{-5}	Significant
**Rp**	F(2, 1.88) = 158.55	0.00807	Significant
**Corrosion rate**	F(2, 1.41) = 2.34×10^{5}	1.27×10^{-4}	Significant

Table S10. Statistical analysis (Games–Howell post hoc) for corrosion parameters of cp-Ti and Ti-xMo (28 and 31 wt.%) samples recorded in in Ringer's solution.

Parameter	Comparison	Mean difference	p-value	Significance
E_{cor}	Ti vs Ti-28Mo	19.67	0.0603	Not significant (trend)
	Ti vs Ti-31Mo	-2.83	0.838	Not significant
	Ti-28Mo vs Ti-31Mo	-22.50	0.249	Not significant
J_{cor}	Ti vs Ti-28Mo	-4.16×10^{-6}	0.110	Not significant
	Ti vs Ti-31Mo	-4.69×10^{-6}	7.35×10^{-5}	Significant
	Ti-28Mo vs Ti-31Mo	-5.25×10^{-7}	0.603	Not significant
**Rp**	Ti vs Ti-28Mo	840,148.47	0.00232	Significant
	Ti vs Ti-31Mo	840,217.26	0.00232	Significant
	Ti-28Mo vs Ti-31Mo	68.79	0.921	Not significant
**Corrosion rate**	Ti vs Ti-28Mo	-0.128	0.110	Not significant
	Ti vs Ti-31Mo	-0.142	3.41×10^{-4}	Significant
	Ti-28Mo vs Ti-31Mo	-0.014	0.647	Not significant
